# Exon and intron sharing in opposite direction-an undocumented phenomenon in human genome-between *Pou5f1* and *Tcf19* genes

**DOI:** 10.1186/s12864-021-08039-6

**Published:** 2021-10-05

**Authors:** Majid Mehravar, Fatemeh Ghaemimanesh, Ensieh M. Poursani

**Affiliations:** 1 Department of Anatomy and Developmental Biology, Development and Stem Cells Program, Biomedicine Discovery Institute, Monash University, Melbourne, Australia; 2 Monoclonal Antibody Research Center, Avicenna Research Institute, ACECR, Tehran, Iran; 3 Hematology, Oncology and Stem Cell Transplantation Research Center, Tehran University of Medical Sciences, Tehran, Iran

**Keywords:** Overlapping genes, Exon sharing, Intron sharing, *Pouf51*, OCT4, *Tcf19*

## Abstract

**Background:**

Overlapping genes share same genomic regions in parallel (sense) or anti-parallel (anti-sense) orientations. These gene pairs seem to occur in all domains of life and are best known from viruses. However, the advantage and biological significance of overlapping genes is still unclear. Expressed sequence tags (ESTs) analysis enabled us to uncover an overlapping gene pair in the human genome.

**Results:**

By using in silico analysis of previous experimental documentations, we reveal a new form of overlapping genes in the human genome, in which two genes found on opposite strands (*Pou5f1* and *Tcf19*), share two exons and one intron enclosed, at the same positions, between OCT4B3 and TCF19-D splice variants.

**Conclusions:**

This new form of overlapping gene expands our previous perception of splicing events and may shed more light on the complexity of gene regulation in higher organisms. Additional such genes might be detected by ESTs analysis also of other organisms.

**Supplementary Information:**

The online version contains supplementary material available at 10.1186/s12864-021-08039-6.

## Introduction

When two genes (either on same strand or opposite strands) share same genomic region(s), then these genes are defined as overlapping genes [1]. Overlapping genes occur frequently in viruses, prokaryotes and eukaryotic mitochondria [2]. The occurrence of overlapping genes in eukaryotic nuclear genome, previously was thought to be rare; however, in recent decades, computational and experimental efforts have started to unveil numerous overlapping genes in eukaryotes, from yeast to plants, insects, and human [3], resulted in a wide acceptance of presence of overlapped genes in spacious genomes such as human genome. Recent reports have shown that ~ 26% of human protein coding genes are overlapping genes [4, 5]. However, the term ‘overlapping gene’ aggregates several related, but different phenomena, including overlaps of introns with introns and exons with introns or exons with exons. Further, in other domains of life, overlapping genes range from short translational couplings of 1-bp overlaps to completely embedded ORFs [2, 6].

The reason for the presence of overlapping genes in the eukaryotic nuclear genome is not clear. As higher eukaryotic genome such as human genome is spacious, occurrence of overlapping genes does not seem to be related to minimizing genome size due to evolutionary pressure. In fact it makes more sense if to be considered as a mechanism of more sophistication regulation of gene expression. In order to better understand the complexity of an organism, understanding the complexity of the genome and its product are inevitable. To reach this goal, moving beyond already annotated RNAs and proteins is needed. Recently research is more appreciating the genomic regions which previously were thought to be junk or outside of annotated regions [6, 7], which may help in better understanding of the genomic architecture and sophisticated gene regulations of higher eukaryotes.

*Pou5f1* encodes for an octamer-binding transcription factor 4A (OCT4A), which binds to octamer motif (ATTTGCAT) of promoter and enhancer of its target genes, and subsequently either accelerates their expression or represses them [8]. OCT4A is highly expressed in pluripotent inner cell mass (ICM) of blastocyst and regulates a wide variety of crucial genes for early stages of development; thus considered as one of the most important stemness markers. As developmental stages proceed further, OCT4A expression becomes restricted to primordial germ cells (PGCs) [9] and very small embryonic-like stem cells (VSELs) [10]. Besides OCT4A, *Pou5f1* gene also is transcribed into multiple other splice variants. Recently we have categorized all known splice variants of OCT4 based on their transcription start sites (TSS) [11]. Briefly four categories of OCT4 splice variants have been identified so far; OCT4A, OCT4B, OCT4C and OCT4D. OCT4B and OCT4C also have multiple alternative splice variants, thus more diversify the mature transcripts of OCT4. The exact function of other splice variants of OCT4 is not fully understood yet; even though some of them are linked to stress conditions such as heat shock [11]. *Pou5f1* gene is located on 6p21.33 (on the minus strand) at a gene dense region flanked by *Psors1c3* and *Tcf19* genes. *Psors1c3* is a non-coding gene located upstream of *Pou5f1* gene on same strand and same orientation, and is involved in immune related disorders and mental disorders [12, 13]. *Tcf19* gene is a protein coding gene that is located on downstream of *Pou5f1* gene on plus strand and in the opposite direction. TCF19 is a transcription factor, and its exact role is not fully understood. It has been reported that this gene is involved in transcription of genes required for late stages of cell cycle progression [14]. It also has been shown that it plays important roles in proliferation of pancreatic β-cells [15], thus its malfunction could be implicated in diabetes [16].

**Fig. 1:**
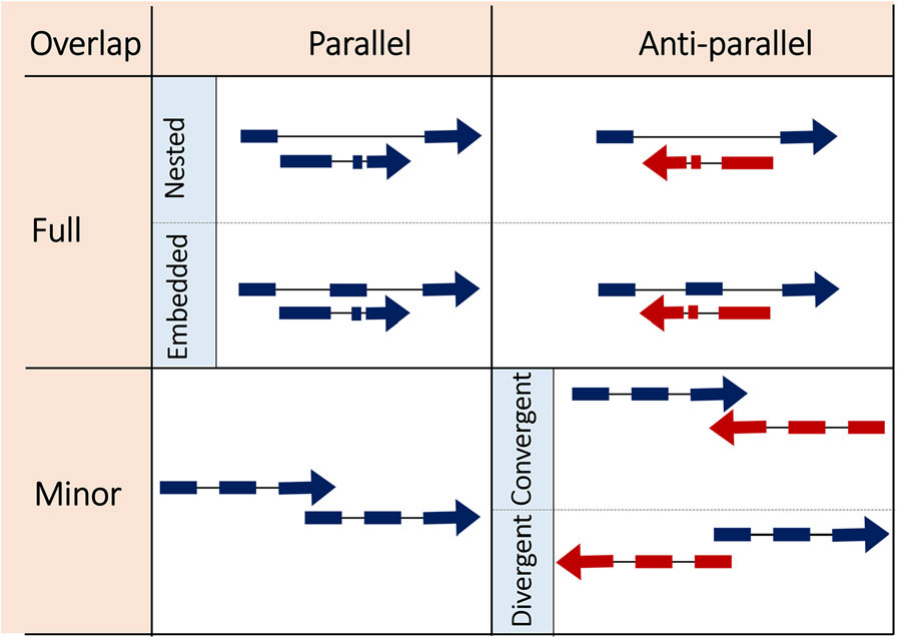
Depiction of different scenarios of gene overlapping architecture. Overlapping genes are either parallel (on same strand) or anti-parallel (on opposite strands). Complete overlap is considered as full, and partial overlap is considered as minor. Full overlap further is categorized into embedded and nested. Minor overlap is either convergent (tail-to-tail) or divergent (head-to-head). *Thin lines represent introns and thick lines represent exons*

Overlapping genes appear in two forms: 1) On same strand or sense-overlapping genes (parallel), 2) On opposite strands or antisense-overlapping genes (anti-parallel). Each category can further be divided into multiple subcategories: full nested, full embedded, partial nested, partial embedded, partial tail-to-tail (partial convergent), partial tail-to-head, partial head-to-head (partial divergent), minor tail-to-tail (minor convergent), minor tail-to-head, and head-to-head (minor divergent) (Fig. 1) [17, 18]. In the present study we report a new form of overlapping gene in the human genome, in which two exons and an enclosed intron of two neighboring genes (*Pou5f1* (*Oct4*) and *Tcf19*) on different strands and opposite directions are shared and, thus, are overlapping, a phenomenon that has not been documented so far.

## Materials and methods

### Data source

OCT4B3 mRNA transcript and TCF19-D related ESTs were downloaded from GenBank-NCBI (https://www.ncbi.nlm.nih.gov/) using “Nucleotide search”. Corresponding transcripts are accessible under accession number KJ624996 (for OCT4B3) and BG192353, BG194808, BG203640, and BG218902 (for TCF19-D related ESTs).

### Sequence visualization

UCSC genome browser (https://genome.ucsc.edu/) was used to visualize *Pou5f1* and *Tcf19* genes. By activating EST tag on UCSC genome browser, all registered ESTs of these two genes were obtained and checked to find overlap regions.

BLAT function was used to browse corresponding sequences; by using details option, exon-intron boundaries were obtained and compared between two different sequences (TCF19-D and OCT4B3).

### Ribosome profiling data analysis

The Genome Wide Information on Protein Synthesis (GWIP-viz) genome browser (https://gwips.ucc.ie/) was used for Ribo-seq data analysis [19]. All studies were included with default parameters for visualization.

### RNA-seq data

Gene expression of *Pou5f1* and *Tcf19* genes in different tissues was obtained from Aceview (https://www.ncbi.nlm.nih.gov/IEB/Research/Acembly/). In addition ENCODE/Caltech RNA-seq was used from UCSC genome browser to obtained RNA-seq reads which are aligned to individual transcripts.

### Comparative analysis

UCSC genome browser was use to visualize the conservation of a particular genomic sequence across 100 different vertebrates. The conservation was calculated based on PhyloP score of each base at particular position. Positive values were sites (nucleotides) that are predicted to be conserved, and negative scored ones are sites (nucleotides) predicted to be fast-evolving.

### Protein prediction

Open Reading Frame (ORF) finder tool implemented in NCBI (https://www.ncbi.nlm.nih.gov/orffinder/) was used to detect any potential open reading frames. ATG was considered to be the start site (start codon) for prediction of potential proteins.

## Results

### OCT4B3 shares two exons and one intron with TCF19-D

**Fig. 2:**
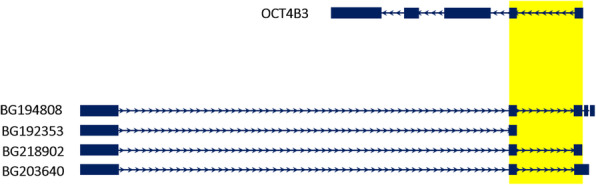
Overlap exons and introns between *Pou5f1* and *Tcf19* genes. OCT4B3 variant (accession number KJ624996) which is a novel variant of *Pou5f1* gene, has overlapping regions with ESTs registered under accession number BG192353, BG194808, BG203640, and BG218902 (all belong to *Tcf19* gene). Overlap regions are highlighted in yellow. *Thin lines represent introns and thick lines represent exons. Arrows show the direction of each gene*

**Fig. 3:**
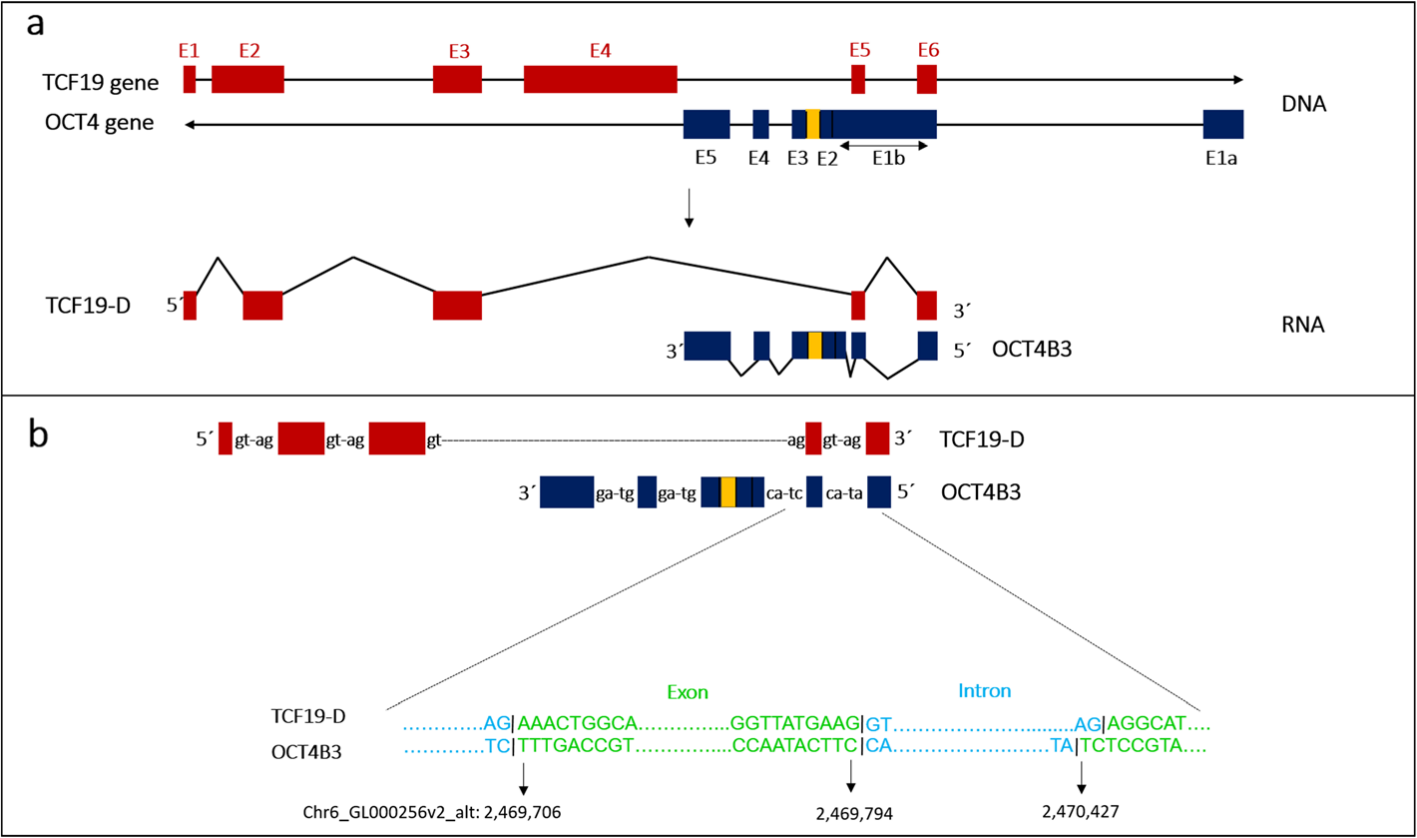
Schematic structure of *Pou5f1* and *Tcf19* genes. TCF19-D variant is transcribed from *Tcf19* gene and OCT4B3 variant is transcribed from *Pou5f1* (*Oct4* gene). TCF19-D and OCT4B3 show overlapped and shared exons and intron (a). Canonical 5’GT-AG3’ splice site is used in TCF19 introns as well as non-overlapped introns of OCT4B3, while overlapped introns of OCT4B3 use non-canonical 5’AT-AC3’ and 5’CT-AC3’ splice site (b). Boxes represent exons and lines between each two boxes represent introns. E: Exon. *Arrows show the direction of each gene. Yellow box is a cryptic exon here, which functions as intron in some of OCT4 transcript* [21]

We have previously reported many splice variants of OCT4 (*Pou5f1*) [11, 20]. Here by using in silico analysis, we have noticed that one of our recently discovered OCT4 novel variants, OCT4B3 (accession number KJ624996), has two exons overlapped with parts of four expressed sequence tags (ESTs) that have been registered on GenBank under accession number-BG192353, −BG194808, −BG203640, and -BG218902 (Fig. 2). Further analysis revealed that these ESTs are transcribed from *Tcf19* gene which is located on plus strand convergent to the *Oct4* gene (Fig. 3a), and designated as TCF19-D. Nucleotide sequences of these ESTs and OCT4B3 were retrieved from GenBank, and then were aligned, which revealed that these two sets of transcripts share two exons (Fig. 3a). Since the shared exons were consecutive, we hypothesized that they must splice out a segment which plays intronic part in mentioned transcripts. Thus, corresponding genomic sequence was retrieved, and corresponding exons and introns were identified, which showed that OCT4B3 and TCF19-D share two exons and one intron enclosed in between, at the same positions (Fig. 3a).

### Canonical and non-canonical splice sites are used in TCF19-D and OCT4B3

Around 99% of mammalian genes use canonical splice site (5’GT-AG3’) to splice out introns [22]. The most frequent non-canonical splice sites include 5’GC-AG3′ and 5’AT-AC3′ with frequency of 0.56 and 0.05%, respectively [22]. Given that OCT4B3 and TCF19-D transcripts share two overlapping exons and an overlapping intron enclosed in between, next we sought to figure out which kind of splice sites are used in these two transcripts. We obtained nucleotide sequences of these transcripts (which contain only exonic parts of corresponding genes) and aligned with human genome (using UCSC genome browser) to figure out intronic parts and their splice sites. Alignment of TCF19-D with the genome showed that all intronic segments of its pre-mRNA contains canonical splice site of 5’GT-AG3′ (Fig. 3b). The same analysis for OCT4B3 was performed, which revealed that the pre-mRNA generating this transcript contains canonical 5’GT-AG3’ splice sites for intron 3 and 4, however splice site for intron 1 contains non-canonical 5’AT-AC3’ sequence and for intron 2 it contains 5’CT-AC3’ sequence (Fig. 3b). By comparing exons and introns of these two transcripts, it is obvious that intron 1 of OCT4B3 is completely overlap and shared with last intron of TCF19-D and also intron 2 of OCT4B3 is spliced out from same position where last to last intron of TCF19-D is spliced out. Taken together, this data shows that TCF19-D and OCT4B3, not only share same exons but also share same introns. And even splice sites of shared introns are complementary to each other in pre-mRNAs of these two transcripts (Fig. 3b).

### Proteins of TCF19-D and OCT4B3

**Fig. 4:**
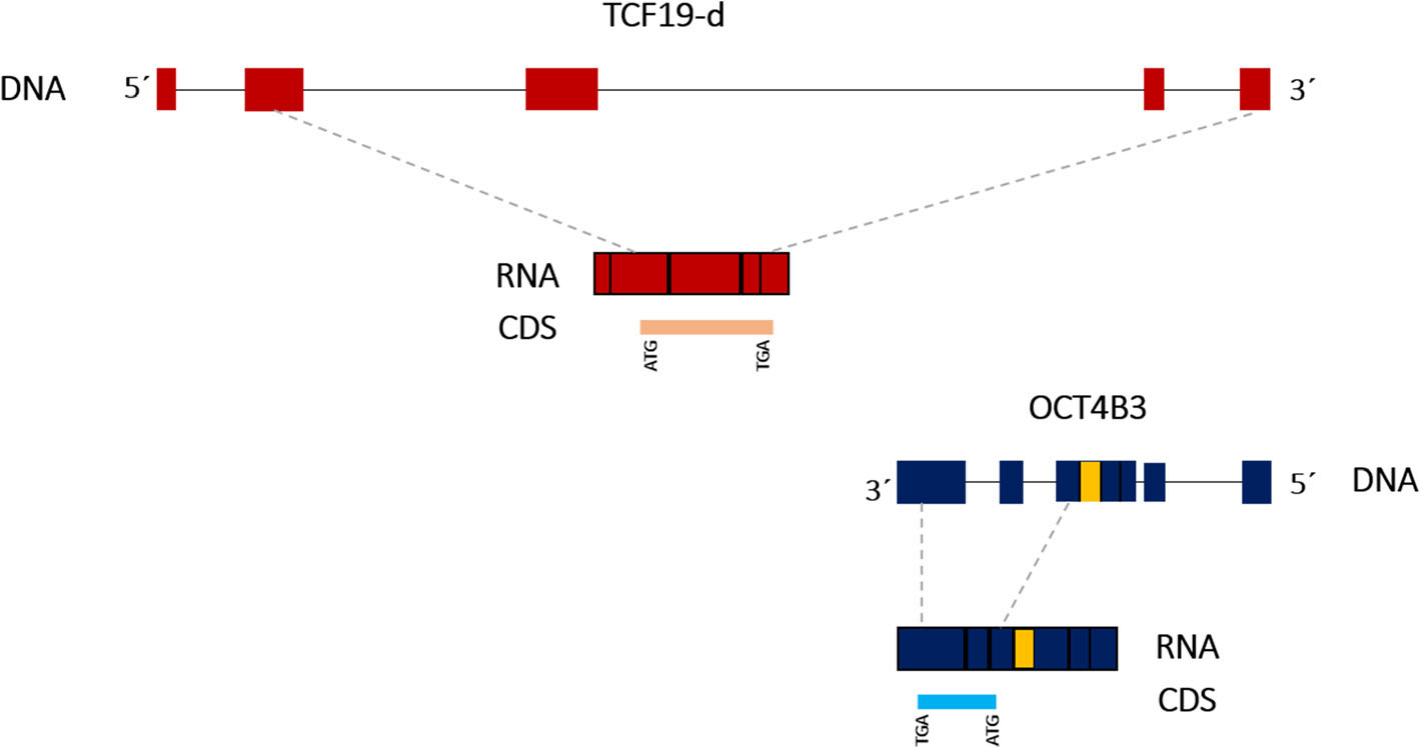
Open Reading Frame (ORF) of TCF19-D and OCT4B3. TCF19-D is predicted to be translated into a 312aa protein (up). Its ORF starts from exon 2 and extends to last exon which is overlap with OCT4B3. OCT4B3 codes a smaller protein with 164aa (down). Predicted ORF is obtained from NCBI ORF finder. Dashed lines show the predicted ORF region. They show start and stop codons and correspond to the region translated

*Tcf19* gene has three validated mRNAs (Variant A, B, and C). All of these mRNAs can code for the same protein with 345 amino acids. Stop codon of their ORFs is located on the last exon which is the longest exon (supplementary Fig. S1). TCF19-D has different exons in its 3’ end compared to variant A, B, and C. The alternative splicing mechanism which generates TCF19-D, splices out the last exon of variant A-C of TCF19 gene (which is the biggest exon and contains stop codon) and introduces two small exons (the shared exons with OCT4B3). Thus, TCF19-D is similar to other variants in beginning of ORF but differs at the end. Last exon of TCF19-D contains stop codon for the same ORF as variant A-C, thus leads to generation of a smaller version of protein (312 amino acids vs 345aa) compare to other variants (Fig. 4). OCT4B3 on the other hand, can encode a 164 amino acids protein [11, 23]. Translation of this protein is internal ribosome entry site (IRES) dependent and thus unlike cap-dependent protein which are reduced under shock or stress condition, its translation remains unaffected under stress conditions [24]. Of note, OCT4B3 ORF is not located on the shared exons. In order to verify the translation of OCT4B3 and TCF19-D, we mined Genome Wide Information on Protein Synthesis (GWIPS-viz) to figure out corresponding ribosome profiling data of human obtained from several studies. Even though we were able to identify the ribosome profiling of TCF19-A, B, and C, however regarding TCF19-D and OCT4 there was no Ribo-seq data obtained from aggregated studies (supplementary Fig. S1). Of note, there was no Ribo-seq data in the aggregated studies for any OCT4 isoform, even though OCT4 is an important stemness factor which is expressed in high level in early embryonic stages and primordial germ cells [25, 26]. Overall, this analysis shows that both TCF19-D and OCT4B3 are predicted to be translated into a putative 312aa and 164aa protein, respectively.

### Gene expression of *Oct4* and *Tcf19*

To compare the expression of Oct4 and Tcf19 in multiple normal tissues we have used RNA-seq gene expression profile of 11 different normal tissues of human (Brain, Ovary, testis, Kidney, Liver, Heart, Skeletal Muscle, Lung, Colon, Lymph Node, and Whole Blood) obtained from the Non-Human Primates Reference Transcriptome Resource (NHPRTR). This resource also contains the expression of selected genes from 14 non-human primates. Based on this data, *Oct4* shows low expression in most of tissues, with the most expression in kidney. However, *Tcf19* is more expressed compared to *Oct4* in tested tissues with the highest expression in lymph node (Supplement Fig. S2). Given this data is obtained from RNA-seq profiling of gene expression which shows the expression of the gene, not specific isoform, then for accurate expression pattern of each isoform, specific quantification was needed. We have previously measured the expression of OCT4B3 in a panel of available cell lines in the lab and published elsewhere [23]. This isoform is expressed in low level in 5637 and 1321 N1 cell lines which are derived from bladder carcinoma and brain astrocytoma, respectively [23]. TCF19-D, on the other hand, has been detected in HT1080 cell line which is a fibrosarcoma derived cell line, as well as thymus [27, 28]. We also mined transcriptome data (RNA-seq) from ENCODE/Caltech projects to figure out the expression of TCF19-D in different cell lines. We noticed that GM78, K562, HUVEC, MCF-7, HCT6, and GM92 cell lines express TCF19-D (Supplementary Table 1).

### Conservation of TCF19-D and OCT4B3

**Fig. 5:**
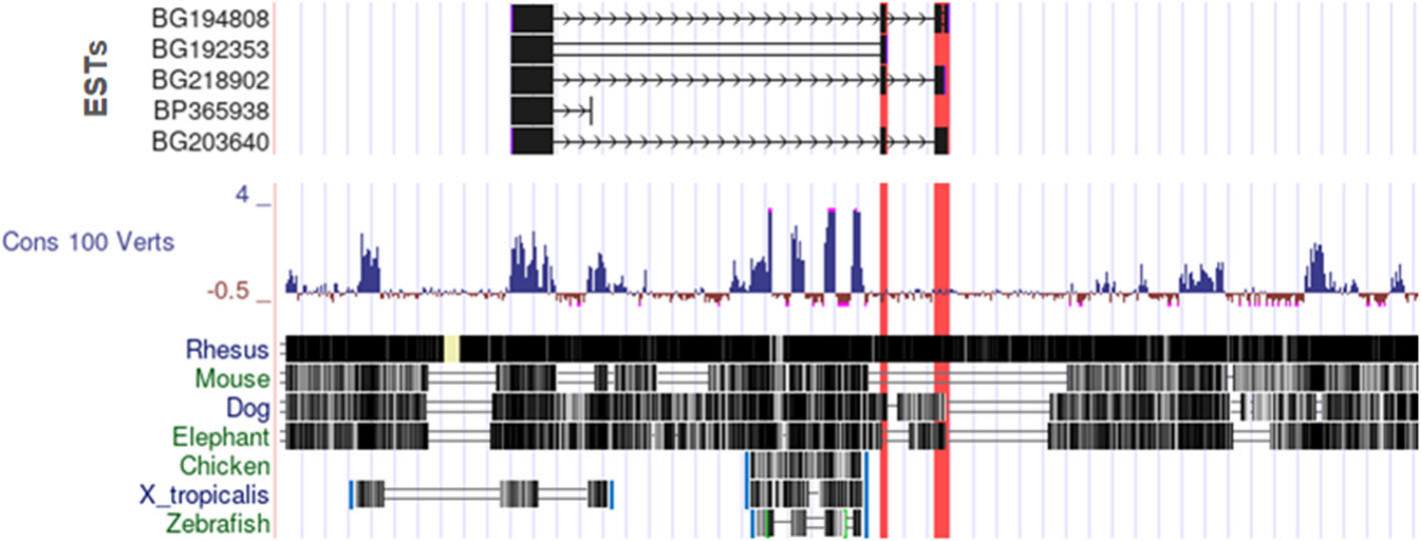
Conservation of *Pou5f1* (*Oct4*) and *Tcf19* gene across different species. Shared exons (highlighted with red) between these two genes do not show any conservation. While, non-overlap exons show significant conservation among vertebrates. The illustration is an screen shot of genomic regions covering *Tcf19* and *Pou5f1* genes (obtained from genome browser-UCSC). The conservation is based on − 0.5 to 4 PhyloP score. Blue area shows conservation and brown area shows negative value which is unique to the species. Those regions that are showing maximum (4) and minimum (− 0.5) scores are capped with pink. Protein coding exons are conserved across species (Shared exons are located out of conserved regions)

During evolution, functional sequences such as protein coding regions and non coding regulatory regions tend to change (evolve) with a slower rate. Thus, comparing a given genomic sequence among species at different evolutionary distances enables us to identify functional regions across species. It also is a useful method to identify unique sequences in a given species [29]. To investigate potential importance of TCF19-D and OCT4B3 across species, we used the UCSC genome browser to evaluate this conservation in 100 vertebrates based on base wise PhyloP score. The main variants of *Oct4* gene (OCT4A) and *Tcf19* gene (TCF19-A) show high positive PhyloP scores (ranging from + 2 to + 4) indicating the conserved functionality of this regions of genome across different species. However, shared regions of OCT4B3 and TCF19-D show PhyloP scores of − 0.5 to + 0.5, which shows that this sequences are unique to the species and do not have evolutionary significance (Fig. 5). Taken together, this data indicates that novel variants of OCT4B3 and TCF19-D are predicted to be fast-evolving species specific regions.

## Discussion

In this study, we have shown that *Pou5f* (*Oct4*) and *Tcf19* genes are overlapping genes. So far, different categories of parallel and anti-parallel overlapping genes have been introduced in a variety of organisms (from single cell organisms to complex organisms), which share same regions on genomic locations [2, 3]. However, our finding reveals a new form of overlapping gene, in which whole exon and whole intron of two adjacent genes in anti-parallel orientation are shared.

In viruses, overlapping genes may endow some advantage due to considerable limitation of genome length; a widely accepted phenomenon, which relatively recently has been challenged by a research group, who demonstrated that overlapping gene can be a convenient way of introducing new gene or reading frame on top of already compact genome of viruses, independent of capsid size or shape [30]. The advantage of overlapping genes in the more complex organisms is less understood [31]. In last decades, large scale expressed sequence tags (ESTs) analysis resulted in discovery of many overlapping transcripts in the human genome; showing ~ 26% of protein coding genes as overlapped [4, 5]. Nevertheless, the exact number of human overlapped genes is still unknown.

When two or more genes are overlapped, sometimes parts of an exon can be shared between them. For example, whole exon3 of *Csec* is a part of exon2 of *Pa17* gene on different strand [32]. Some overlapping genes appear as different ORFs on same genomic regions. For instance, *Polg* gene located on chromosome 15 which encodes for DNA polymerase γ (Pol γ), responsible for mitochondrial DNA repair and replication, appeared to be a parallel overlapping gene which has three different ORFs (Pol γ, ORF-Y, ORF-Z), each codes for different proteins [33].

Here, for the first time, we report a type of sense-antisense overlapping phenomenon in which two separate convergent neighboring genes on the opposite strands share same exons and splice out same intron in some of their splice variants.

Both OCT4B3 [11] and TCF19-D are predicted to be protein coding. However, ORF of OCT4B3 is not in the overlapping region, and ORF of TCF19-D is extended to the overlapping region. In addition, shared regions of these variants are not conserved through evolution, which emphasizes that this overlapping may be related to fast evolving features which is important for sophistication of higher organisms.

The biological significance of sense-antisense overlap is not clear, but it is suggested that transcripts that share exons may have regulatory roles on each other by binding to each other on their overlapped reverse complement sequences. This kind of RNA-RNA base pairing may play important regulatory roles as evidenced by the fact that sense-antisense dsRNA structures contribute to many mammalian gene-regulatory phenomena [34] including RNA degradation [35], RNA splicing, DNA methylation, genomic imprinting, masking of miRNA binding sites and miRNA sponge [36]. Deciphering whole exon and whole intron sharing from opposite directions in the human genome adds a new layer to splicing process and may contribute to the complexity of higher eukaryotes which warrants further investigations.

In conclusion, this study demonstrates that whole exon and whole intron sharing on different strands of DNA with opposite directions occurs in the human genome, which requires more comprehensive studies to realize its exact regulatory functions.

## Supplementary Information

**Additional file 1.**

## Data Availability

All online tools, software and data bases which have been used in this study are freely available. Refer to the Material and Method section for specific links. Briefly NCBI, UCSC genome browser, GWIP-vis, Aceview, and NCBI ORF finder can be accessed at https://www.ncbi.nlm.nih.gov/, https://genome.ucsc.edu/, https://gwips.ucc.ie/, https://www.ncbi.nlm.nih.gov/IEB/Research/Acembly, and https://www.ncbi.nlm.nih.gov/orffinder/ respectively. Accession number for OCT4B3 is KJ624996; ESTs of TCF19-D can be accessed using following accession numbers: BG192353, BG194808, BG203640, and BG218902.
