# Supplementary material for: Exon and intron sharing in opposite direction-an undocumented phenomenon in human genome-between Pou5f1 and Tcf19 genes
Source: BMC Genomics. 2021 Oct 5;22:718. doi: 10.1186/s12864-021-08039-6 (PMC8493703; doi:10.1186/s12864-021-08039-6)
Supplement: Supplementary file 1 — Additional file 1. [file 12864_2021_8039_MOESM1_ESM.docx]

**Supplementary File**

**Figure S1. Ribo-seq profiling (obtained from several studies) showing coverage of Ribo-seq reads on TCF19 and OCT4 locus.** TCF19 A, B, and C variants have same ORF and show considerable Ribo-seq read coverage (left). OCT4 (right) does not show any significant coverage (even though it is a protein coding gene (we assumed that it has not been captured by corresponding studies)). TCF19-D which extends to *OCT4* gene is not shown (because the Genome browser only shows previously annotated variants).

**Figure S2. Gene expression of *Pou5f1* and *Tcf19* in different tissues at the level of RNA.** RNA-seq data obtained from Non-Human Primates Reference Transcriptome Resource (NHPRTR) project.

Supplementary Figure S1


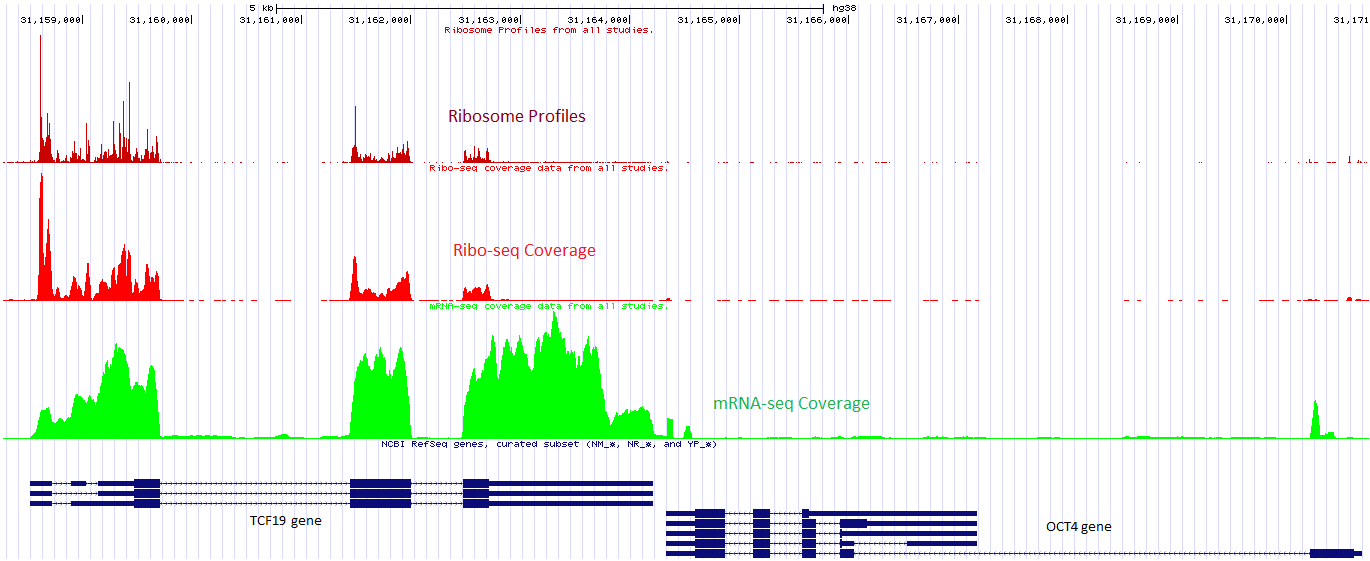


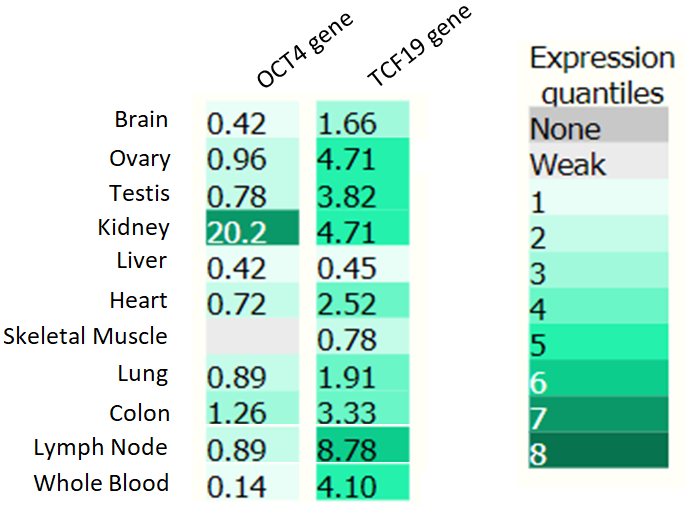
Supplementary Figure S2

Supplementary Table 1. Number of RNA-seq reads overlapped with TCF19-D (obtained from ENCODE/Caltech project).

| Cell line | Origin | Number of overlapped reads |
| --- | --- | --- |
| GM78 | Mammary gland cell line | 16 |
| K562 | Chronic myelogenous leukemia | 6 |
| HUV-EC-C | Umbilical vein/vascular endothelium | 8 |
| MCF-7 | Breast cancer | 2 |
| HCT116 | Bone marrow | 4 |
| GM92 | mammary gland cell line | 9 |
